# Echocardiographic Left Ventricular Mass Assessment: Correlation between 2D-Derived Linear Dimensions and 3-Dimensional Automated, Machine Learning-Based Methods in Unselected Patients

**DOI:** 10.3390/jcm10061279

**Published:** 2021-03-19

**Authors:** Andrea Barbieri, Francesca Bursi, Giovanni Camaioni, Anna Maisano, Jacopo Francesco Imberti, Alessandro Albini, Gerardo De Mitri, Francesca Mantovani, Giuseppe Boriani

**Affiliations:** 1 Division of Cardiology, Department of Diagnostics, Clinical and Public Health Medicine, Policlinico University Hospital of Modena, University of Modena and Reggio Emilia, 41121 Modena, Italy; olmoberg@libero.it (A.B.); camaioni.giovanni@unimore.it (G.C.); anna.maisano@unimore.it (A.M.); jacopo.imberti@unimore.it (J.F.I.); alessandro.albini@unimore.it (A.A.); gerardo.demitri@unimore.it (G.D.M.); giuseppe.boriani@unimore.it (G.B.); 2 Division of Cardiology, San Paolo Hospital, ASST Santi Paolo and Carlo, Department of Health Sciences, University of Milan, 20142 Milan, Italy; Bursi@libero.it; 3 Cardiology, Azienda USL-IRCCS di Reggio Emilia, 42123 Reggio Emilia, Italy

**Keywords:** 2D echocardiography, 3D echocardiography, left ventricular mass, machine learning

## Abstract

A recently developed algorithm for 3D analysis based on machine learning (ML) principles detects left ventricular (LV) mass without any human interaction. We retrospectively studied the correlation between 2D-derived linear dimensions using the ASE/EACVI-recommended formula and 3D automated, ML-based methods (Philips HeartModel) regarding LV mass quantification in unselected patients undergoing echocardiography. We included 130 patients (mean age 60 ± 18 years; 45% women). There was only discrete agreement between 2D and 3D measurements of LV mass (r = 0.662, r^2^ = 0.348, *p* < 0.001). The automated algorithm yielded an overestimation of LV mass compared to the linear method (Bland–Altman positive bias of 13.1 g with 95% limits of the agreement at 4.5 to 21.6 g, *p* = 0.003, ICC 0.78 (95%CI 0.68−8.4). There was a significant proportional bias (Beta −0.22, t = −2.9) *p* = 0.005, the variance of the difference varied across the range of LV mass. When the published cut-offs for LV mass abnormality were used, the observed proportion of overall agreement was 77% (kappa = 0.32, *p* < 0.001). In consecutive patients undergoing echocardiography for any indications, LV mass assessment by 3D analysis using a novel ML-based algorithm showed systematic differences and wide limits of agreements compared with quantification by ASE/EACVI- recommended formula when the current cut-offs and partition values were applied.

## 1. Introduction

The quantification of the left ventricular (LV) mass by echocardiography is based on detracting the volume of the LV cavity from the volume enclosed by the corresponding epicardium to obtain myocardial volume and then multiplying by myocardial density (taken at 1.05 g/mL) [1].

The linear method for assessing LV mass is the historical reference norm because of its simplicity and wide availability, despite having significant limitations related to the need for cardiac geometric assumptions of the prolate ellipsoid [2]. Besides, previous comprehensive population studies have shown that quantification of LV mass by the 2D-derived linear dimensional system and partition values as suggested by the American Society of Echocardiography/European Association of Cardiovascular Imaging (ASE/EACVI) [3] has robust event predictive power in adults undergoing echocardiography for any indication [4,5]. 

Linear methods, however, are vulnerable to small measurement errors due to the need to calculate myocardial volume by cubing linear dimensions related to accuracy and reproducibility compared to cardiac magnetic resonance [6,7,8]. Conversely, 3D is the only echocardiographic technique that directly analyses myocardial volume, without anatomical assumptions of LV form and wall thickening distribution. This technique is therefore promising and can be used in abnormally shaped ventricles. Improvement in 3D technology provided a new algorithm for 3D LV mass analysis, which based on the principles of machine learning (ML) (Dynamic Heart Model (DHM), Philips Healthcare, Andover, MA, USA) using a training set of over a thousand studies was validated against cardiac magnetic resonance for the evaluation of LV size and function [8,9,10,11]. Left ventricular epicardial borders are also detected by the current fully automated version of the ML software without the need for manual tracing, allowing for a feasible, fast, accurate, and reproducible automated quantification of LV mass [12].

Therefore, applying artificial intelligence or ML techniques to 3D has the great potential to revolutionize how we diagnose and quantify LV mass. However, data obtained from automated ML-based software must be incorporated into actual practice as the existing ASE/EACVI-approved cut-off values based on the 2D-derived linear dimension methodology may not extend to the 3D automated, ML-based generation. Accordingly, given the significant clinical implications, we sought to test the potential impact of this technology, assessing the correlation between 2D-derived linear dimensions and 3D automated, machine learning-based methods regarding LV mass quantification in consecutive patients undergoing echocardiography for any indications.

## 2. Materials and Methods

The study population comprised unselected elective in- and outpatients, aged more than 18 years-old, who underwent standard Doppler echocardiography for any indication from June 2020 to September 2020 at Modena University Hospital’s echocardiography laboratory. Criteria for enrollment included (1) age >18 years and (2) complete resting 2D and 3D echocardiographic assessment, including real-time LV mass measurement. We excluded all conditions in which the geometric assumptions inherent in the 2D-linear approach remain problematic: (1) patients with gross shape deformations, such as that caused by post-myocardial infarction focal remodeling, (2) the presence of discrete upper septal or asymmetric hypertrophy, and (3) other diseases with gross regional variations in wall thickness (i.e., hypertrophic cardiomyopathy, ≥two contiguous segments with scar tissue).

Then, 3D and 2D imaging were performed on the same exam. LV mass was measured from 3D images using the novel automated software DHM algorithm and compared against the 2D-derived linear dimensions, guideline-recommended reference technique. Age, sex, height, weight, BSA, cardiac rhythm, clinical indications, and history of cardiovascular diseases were recorded at the time of echocardiography.

### 2.1. Echocardiographic Data

A complete 2D echocardiographic examination was performed, according to current guidelines [3,13], using a commercial ultrasound system (EPIQ CVx, Philips Healthcare) equipped with an X5−1 transducer. LV diameters and septal and posterior wall thickness were measured using the linear 2D method from the parasternal long-axis view. The use of 2D-derived linear dimensions overcomes the common problem of overestimating the M-mode’s cavity and wall dimensions due to oblique scan in parasternal images. LV end-diastolic dimensions were measured at the onset of the QRS complex at the LV minor dimension level at the mitral leaflet tips level. In patients with atrial fibrillation, we used a single-beat acquisition mode and multiple cardiac cycles.

**Figure 1:**
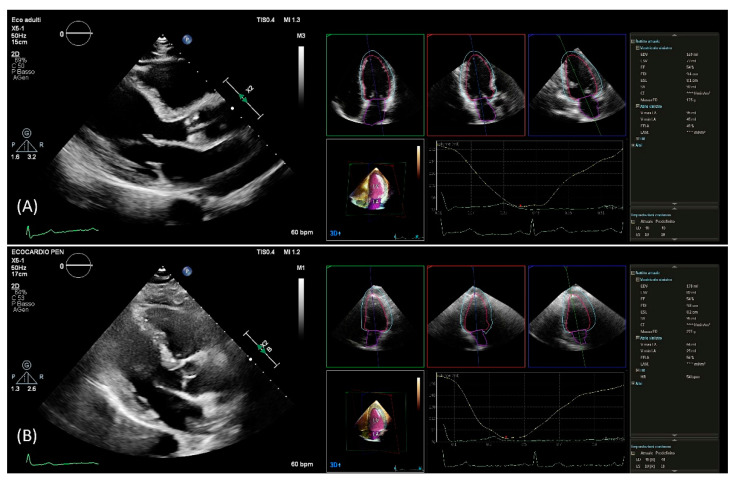
PLAX view used for linear 2D measurements and 3D measurements obtained from automated DHM software in a patient with good agreement (**A**) and in a patient with poor agreement (**B**) between 2D and 3D mass values. DHM, Dynamic Heart Model; PLAX, parasternal long axis; 2D, two-dimension; 3D, three-dimension.

After setting gain, time-gain compensation, and depth on 2D images, a single-beat acquisition mode from the apical 4-chamber view was used to acquire 3D wide-angle datasets. By changing sector width and image depth, the 3D frame rate was optimized. All measurements were performed by the same operator (AB), fully trained in echocardiography with long-standing experience with the 3D technique and trained on echocardiographic datasets with the focus on what constitutes adequate automated analysis. Briefly, the novel vendor software simultaneously detects LV and left atrial endocardial surfaces using an adaptive analytics algorithm, which uses knowledge-based identification to orient and locates cardiac chambers and patient-specific adaptation of endocardial borders from which LV and left atrial volumes are derived directly without geometrical assumptions (Figure 1). LV volumes, left atrial volumes, and ejection fraction were assessed using the 3D method. All measurements were performed online and entered into an electronic database at the time of the echocardiographic study. No modification from the original database was applied, and no measurement was made offline. Hence, the study consisted of a retrospective analysis of data entered into the electronic echocardiographic database. 

### 2.2. Left Ventricular Mass Quantification 

2D-LV mass was obtained using the ASE/EACVI-recommended formula for the estimation of LV mass from LV linear dimensions based on modeling the left ventricle as a prolate ellipse of revolution: LV mass (g) = 0.8(1.04(LVIDD + IVST + PWT)3 LVIDD3) + 0.6, where LVIDD is LV internal end-diastolic dimension, IVST is the end-diastolic interventricular septal wall thickness, and PWT is end-diastolic LV posterior wall thickness [2].

Using the automated DHM program, which automatically detects LV endo- and epicardial borders at the end-diastole, 3D-LV mass was analyzed, enabling direct LV mass quantification (Figure 1). While it is possible to correct the LV and left atrial endocardial surfaces manually, no changes to the automatically identified cardiac boundaries have been made. Previous studies have shown that LV volumes [8,9,14] and LV mass [12] could be accurately measured using this software, and manual border adjustments led to only clinically insignificant differences. In this study, 3D echocardiography images were analyzed using the same boundary detection sliders for all patients (40 for end-diastole and 10 for end-systole). 

The quality of the 2D echocardiographic images was assessed in both the parasternal short-axis and the apical four-chamber views and classified into three categories: (1) good images—the endocardial and epicardial boundaries were well defined on both short-axis and apical four-chamber images in end-diastole and end-systole, (2) sufficient images—the endocardial and epicardial margins displayed a partial echocardiographic dropout area (less than 1/8 of the total circumference), and (3) unsatisfactory—the margins were not seen well and were thus deemed to be untraceable. The unsatisfactory group was excluded from further analysis. 

To assess the intra-observer reproducibility of the new 3D algorithm, repeated measurements were performed in a subsequent cohort of patients by the same observer blinded to all prior measurements. The institutional review boards approved the study of our institution.

### 2.3. Statistical Analysis 

Data are shown as percentages for categorical variables and as mean ± SD for continuous variables. Comparisons across groups were made using c2 tests for categorical variables and variance analysis for continuous variables or Kruskal-Wallis tests for highly skewed variables. The inter-technique agreement was tested using Pearson’s linear correlation and Bland–Altman analysis [15]. The Cohen kappa statistics were used to calculate the strength of the accord in categorizing LV mass by 2D and 3D. The percent of the agreement was calculated as the ratio between agreed-on measures and the total. Intra-observer variability was assessed using the interclass correlation coefficient (ICC) and coefficient of variation (CoV). All tests were two-tailed. *p* values < 0.05 were considered statistically significant. All analyses were performed using SPSS version 15.0 for Windows (SPSS, Inc., Chicago, IL, USA). 

## 3. Results

### 3.1. Patients’ Characteristics

**Figure 2:**
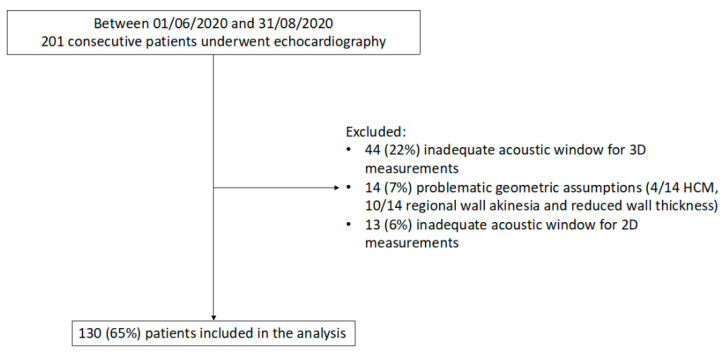
Flow diagram showing inclusion/exclusion criteria for the study population. HCM, Hypertrophic cardiomyopathy; 2D, two-dimensional, 3D, three-dimensional.

Of the 201 consecutive patients who underwent echocardiography in the study period, 44 (22%) were excluded because of an inadequate acoustic window for 3D measurements, 14 (7%) for problematic geometric assumptions, and 13 (6%) due to insufficient acoustic window for 2D measurements (Figure 2). Thus, the study included 130 patients (mean age 60 ± 18 years; 45% women) with a wide range of LV mass and study indications (Table 1 and Table 2). Of note, significant differences were found in LV mass quantification between 2D and 3D (*p* = 0.003) with a mean LV mass by 2D of 158 ± 64 g and 171 ± 53 g by 3D (Table 2). 

### 3.2. Agreement Analysis

**Figure 3:**
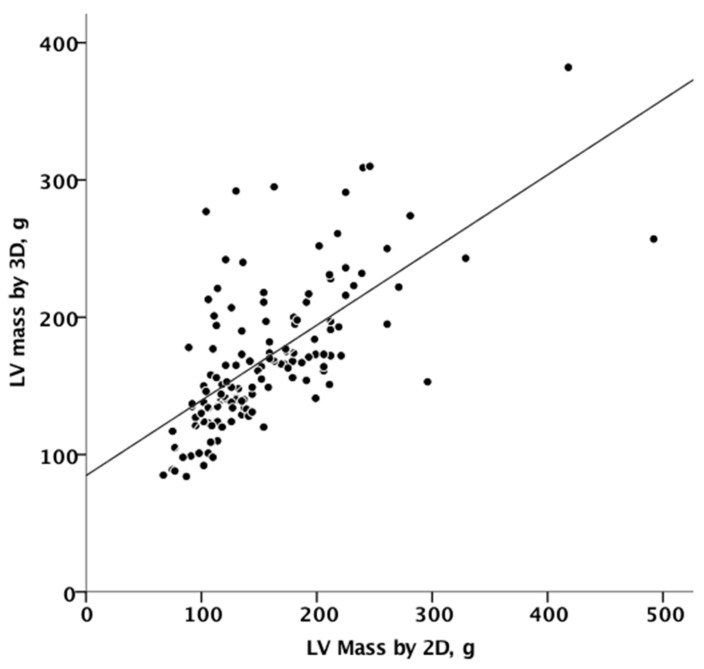
Linear correlation between 2D and 3D measurements of mass. 2D, two-dimensional; 3D, three-dimensional.

**Figure 4:**
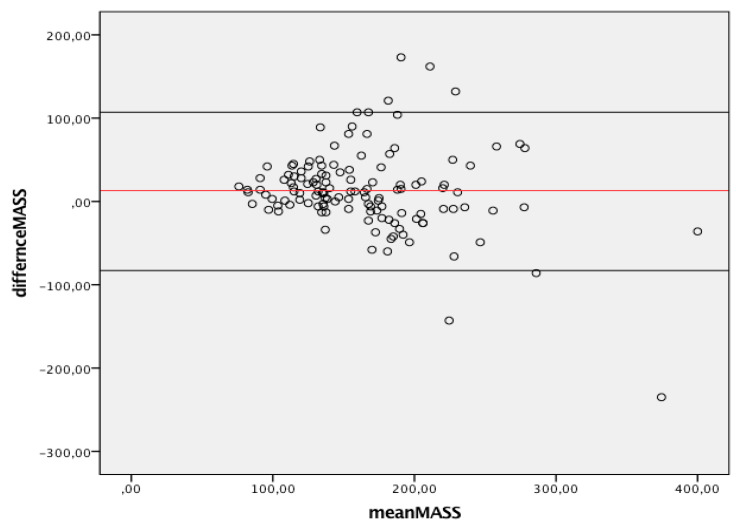
Bland-Altman graph. Y axes depicts difference between 2D and 3D same operator measured mass while X axis represents the average of measurements. Solid red line indicates average difference while black solid lines indicate 95%CI. CI, Confidence interval; 2D, two-dimensional; 3D, three-dimensional.

Figure 3 summarizes the agreement between linear and 3D measurements of LV mass, showing only discrete agreement, as evidenced by Pearson’s correlation (r = 0.662, r^2^ = 0.348, *p* < 0.001), and the results of Bland–Altman analysis (Figure 4). The automated algorithm resulted in an overestimation of LV mass compared to the linear method, reflected by a significant positive bias of 13.1 g (7.6% of the mean measured value) with 95% limits of the agreement at 4.5 to 21.6 g, *p* = 0.003, ICC 0.78 (95%CI 0.68−8.4). There was a significant proportional bias (Beta −0.22, t = −2.9), *p* = 0.005, regarding the variance of the difference across the range of LV mass. Of note, the vertical scatter of data was narrower for lower values of mass. 

The results were consistent across subgroups. Mainly when the exam was performed for screening, r = 0.765 *p* < 0.001, when it was performed for any other indication r = 0.637, *p* < 0.001. When the exam was performed to analyze VHD r = 0.612 *p* < 0.0001. Among patients with hypertension r = 0.803, *p* < 0.0001, the correlation coefficient among those without hypertension was r = 0.572, *p* < 0.0001. Among women r = 0.679, among men r = 0.593, *p* < 0.001. Other groups less represented were not analyzed.

### 3.3. Mass Categries Adjudication

When the published cut-offs for LV mass abnormality were used, 24 patients (18.5%) were classified as having abnormal mass by 2D and 27 (20.7%) by 3D (*p* < 0.001). Of the 24 who had abnormal mass by 2D, 10 patients (41.6%) had normal mass by 3D. Conversely, of the 27 with abnormal 3D mass, 13 had normal mass by 2D (48.1%). The observed proportion of overall agreement was 82% (kappa = 0.44, *p* < 0.001), Table 3.

Table 4 shows the different distributions of LV mass categories applying 2D-linear vs. 3D measurements. The observed proportion of overall agreement was 77% (kappa = 0.32, *p* < 0.001). 

### 3.4. Reliability Analysis

The reliability analysis was performed in a different group of 48 random patients: the mean difference between the measurements obtained by a single reader was 0.81 ± 8.7 g, and the interclass correlation coefficient was 98%. When the window was good (27 pts), the mean difference between the measurements obtained was 1.0 ± 6.0 g, and the interclass correlation coefficient was 99%; for sufficient windows (21 patients), the intraobserver mean difference was 0.5 ± 11 g, and the interclass correlation coefficient was 97%, both indicating outstanding reliability.

Intraobserver variability coefficients were 2.6 ± 2.5%; particularly 2.3% ± 2.5 for patients with good windows, 2.9% ± 2.4 for sufficient window.

## 4. Discussion

It is easy to predict that, with artificial intelligence development, echocardiographic diagnostic practice will change radically very soon. In this context, the 3D ML automated approach to left-heart chamber quantification based on an adaptive analytics algorithm represents a rapidly evolving approach to LV mass quantification. It can potentially handle LV volume changes and, therefore, a better alternative to the linear method [16]. 

However, the open question is, what will happen in our laboratories if ML techniques could eventually supersede the traditional linear method for LV mass quantification?

Studies comparing 2D-guided linear LV mass measurements with 2D echocardiographic area-length or truncated ellipsoid methods in usually shaped ventricles have shown small differences [1]. Still, related documentation with fully automated 3D analysis has not been carried out in the present period. Therefore, the current work poses four critical and original clinical concerns.

First, our data show that image quality remains a significant contributor to LV mass measurement feasibility when fully automated 3D processing is performed. Indeed, roughly 20% of patients had insufficient quality images. This proportion reflects the real-life clinical feasibility because it included a cohort of unselected patients yet routinely seen in clinical practice not enrolled in previous studies. Low and fair-quality images have been found to increase erroneous machine border monitoring, and automatic software has not worked in some patients. Contour modifications can improve automated analysis accuracy, but this increases the time of examination and decreases the workflow’s efficiency. Therefore, by defining patient subsets that should be included and excluded from 3D automated quantification, each operator should set personal standards [17].

Second, our findings indicate systematic variations between linear and 3D echocardiography approach with a mean difference of 13.1 g among the two methods. Furthermore, the comparison between automated 3D and linear measurements showed wide limits of agreements. This finding emphasizes the potential real-world variability of echocardiographic LV mass quantification. 

Although the ASE/EACVI-recommended formula should be reported in all echocardiograms performed in patients without “major LV remodeling” [13], but these conditions are not outlined. LV mass quantification by the linear method is prone to wrongly changes according to changes in LV volume. For example, the large fluid shift in hemodialysis patients, which causes significant differences in end-diastolic LV internal diameters, apparently affects LV mass calculations by linear methods, but not by the 2D echocardiographic area-length method [18].

Third, the misclassification between the linear and automatic 3D methods was magnified when cut-offs are applied since only 77% of the cases were classified in the same way.

One limitation to 3D LV mass reporting is that there are currently no age- or gender-specific reference cut-offs for interpretation. It should also be noted that continuous improvements in 3D echocardiographic imaging’s spatial and temporal resolution will also influence normal values. Depending on the manufacturer and the version of the echocardiography machines being used, the types of automated quantification techniques available will vary.

Fourth, we confirmed that one of the main benefits of 3D automated quantification is its intra-observer reproducibility. We found that the mean difference between the measurements obtained by a single reader was 0.81 ± 8.7 g and the interclass correlation coefficient was 98%. For particular clinical conditions requiring higher precision and reproducibility, automated 3D LV mass quantification will be preferable. An essential benefit of the new algorithm is that it automatically performs the analysis. Small cardiac boundary changes require less skill than manually mapping the boundaries without any hints, reducing professional and inexperienced readers’ distance [19].

## 5. Limitations

Independent verification from a core laboratory or the use of a gold standard like cardiac magnetic resonance was not reported and could perhaps help us understand the accuracy and precision of this new 3D technology. However, Volpato et al. recently showed that the LV mass quantification using the same novel automated software DHM algorithm this ML-based algorithm is very similar to cardiac magnetic resonance-derived values (Bland–Altman bias 5 g, limits of agreement ±37 g) [12].

We only performed intra-analysis on the 3D echocardiograms. This choice was made specifically to avoid introducing additional bias in our initial experience with this 3D automatic LV mass quantification. 

In our study, we have arbitrarily chosen the default settings of boundary detection sliders, designed to globally increase or decrease the size of the endocardial 3D surface, at the end-diastolic default position = 40/40, since lead to images that are closer to those obtained with 2D [9]. Indeed, our aim was to further decrease potential bias in head-to-head 2D−3D comparison. Nevertheless, when the laboratory policy strengthens the correlation between automatic 3D and the conventional validated 2D method, more studies are needed to define the theoretical ideal automatic slider position.

The agreement between linear and 3D measurements of LV mass may have been overestimated as 7% of patients were excluded for problematic geometric assumptions of the 2D-derived linear dimensions method. However, LV mass assessment could theoretically benefit from 3D automated method analysis, especially in patients with the excluded group’s characteristics.

Finally, only 10% of our patients had a sufficient acoustic window, while 90% had an excellent acoustic window. However, the fact that we did not detect a significant difference in intra-observer reproducibility between patients with sufficient and good acoustic windows is reassuring.

## 6. Conclusions

In consecutive patients undergoing echocardiography for any indication, the 3D LV mass assessment using a novel ML-based algorithm showed systematic discrepancies and large limits of agreements compared to the ASE/EACVI-recommended formula when the current cut-offs and partition values were applied.

## Figures and Tables

**Table 1:** Baseline clinical characteristics of the study population.

	*N* (%) or Mean ± SD
Age	60 ± 18
Female sex	58 (44.6%)
***Reasons for the exam and comorbidities***	
Screening	27 (20.8%)
Dyspnea	12 (9.2%)
Suspicion of bacterial endocarditis	8 (6.2%)
Chemotherapy	25 (19.2%)
Coronary artery disease	9 (6.9%)
Acute decompensated heart failure	2 (1.5%)
Dilated cardiomyopathy	8 (6.2%)
Valve heart disease	32 (24.6%)
Hypertension	39 (30.0%)
Diabetes mellitus	12 (9.2%)
Follow-up of previous myocarditis or pericarditis	5 (3.8%)
Pulmonary embolism	1 (0.8%)
Atrial fibrillation	22 (16.9%)
COPD	6 (4.6%)
Stroke or TIA	9 (6.9%)
Dialysis	10 (7.7%)
Tumor	38 (29.2%)
Severe liver disease	6 (4.7%)

*N*, number; SD, Standard deviation; COPD, Chronic obstructive pulmonary disease; TIA, transient ischemic attack.

**Table 2:** Echocardiographic characteristics of the study population.

	N (%) or Mean ± SD
***Echocardiographic exam***	
Rhythm during echocardiogram:	
Sinus	114 (87.7%)
Atrial fibrillation	14 (10.8%)
Pacemaker	2 (1.5%)
Image quality:	
Good	117 (90%)
Sufficient	13 (10%)
Heart rate during echocardiogram, bpm	73 ± 15
BSA, m^2^	1.8 ± 0.2
Maximal left atrial volume by 3D, mL	71 ± 35
Lef atrial ejection fraction by 3D, %	56 ± 16
End-diastolic LV diameter by 2D, mm	50 ± 6
Indexed LV end-diastolic diameter by 2D, mm/m^2^	28 ± 3
End-diastolic LV volume by 3D, mL	123 ± 43
Indexed end-diastolic LV volume by 3D, mL/m^2^	67 ± 20
Systolic LV volume by 3D, mL	50 ± 29
Indexed systolic LV volume by 3D, mL/m^2^	27 ± 15
LV ejection fraction by 3D, %	61 ± 10
SV by 3D, mL	74 ± 22
Interventricular septal thickness by 2D, mm	8.8 ± 1.7
Posterior wall thickness by 2D, mm	8.7 ± 1.7
LV mass by 2D, g	158 ± 64
Indexed LV mass by 2D, g/m^2^	86 ± 32
Relative wall thickness	0.35 ± 0.06
LV mass by 3D, g	171 ± 53
Mitral regurgitation:	
Absent/trivial	105 (81%)
Mild	15 (11%)
Moderate	5 (4%)
Severe	5 (4%)
Aortic regurgitation:	
Absent/trivial	116 (89%)
Mild	9 (6.9%)
Moderate	3 (2.3%)
Severe	2 (1.5%)

*N*, number; SD, Standard deviation; BSA, body surface area; 3D, three-dimension; LV, left ventricle; 2D, two-dimension; SV, stroke volume.

**Table 3:** Number of normal and abnormal indexed lass values measured by 2D and 3D.

	Normal Indexed Mass by 3D *N* = 103	Abnormal Indexed Mass by 3D *N* = 27
**Normal indexed mass by 2D** ***N* = 106**	93	13
**Abnormal indexed mass by 2D** ***N* = 24**	10	14

2D, two-dimension; 3D, three-dimension.

**Table 4:** Different distributions of LV mass categories applying 2D-linear vs. 3D measurements. The observed proportion of overall agreement was 77% (kappa = 0.32, *p* < 0.001).

	Normal iLV Mass by 3D *N* = 103	Mildly Abnormal iLV Mass by 3D *N* = 11	Moderately Abnormal iLV Mass by 3D *N* = 8	Severely Abnormal iLV Mass by 3D *N* = 8
**Normal iLV mass by 2D** ***N* = 106**	93	8	4	1
**Mildly abnormal iLV mass by 2D** ***N* = 12**	8	3	0	1
**Moderately abnormal iLV mass by 2D** ***N* = 3**	1	0	0	2
**Severely abnormal iLV mass by 2D** ***N* = 9**	1	0	4	4

LV, Left ventricular; 2D, two-dimension; 3D, three-dimension.

## Data Availability

Data available on request due to restrictions (e.g., privacy or ethical).
